# Periodontal Pathogens and Preterm Birth: Current Knowledge and Further Interventions

**DOI:** 10.3390/pathogens10060730

**Published:** 2021-06-09

**Authors:** Milan Terzic, Gulzhanat Aimagambetova, Sanja Terzic, Milena Radunovic, Gauri Bapayeva, Antonio Simone Laganà

**Affiliations:** 1 Department of Medicine, School of Medicine, Nazarbayev University, Nur-Sultan 010000, Kazakhstan; milan.terzic@nu.edu.kz (M.T.); sanja.terzic@nu.edu.kz (S.T.); 2 Clinical Academic Department of Women’s Health, National Research Center of Mother and Child Health, University Medical Center, Nur-Sultan 010000, Kazakhstan; gauri.bapayeva@gmail.com; 3 Department of Obstetrics, Gynecology and Reproductive Sciences, School of Medicine, University of Pittsburgh, Pittsburgh, PA 15213, USA; 4 Department of Biomedical Sciences, School of Medicine, Nazarbayev University, Nur-Sultan 010000, Kazakhstan; 5 Laboratory for Microbiology, School of Dental Medicine, University of Belgrade, 11000 Belgrade, Serbia; milena.radunovic@stomf.bg.ac.rs; 6 Department of Obstetrics and Gynecology, “Filippo Del Ponte” Hospital, University of Insubria, 21100 Varese, Italy; antoniosimone.lagana@uninsubria.it

**Keywords:** periodontal pathogens, preterm labor, preterm birth, periodontal disease, pregnancy

## Abstract

Preterm labor is defined as a birth before 37 weeks of gestation and occurs in 5–20% of pregnancies. Preterm labor, as multifactorial entity associated with a high risk of neonatal morbidity and mortality, is influenced by maternal, fetal and environmental factors. Microbiological studies suggest that infectious pathogens may account for 25–40% of preterm birth. Infections of different sites, like genital, urinary tract infections, and pneumonia, are linked to the preterm labor. The most recent epidemiological studies consistently report that maternal periodontal disease is associated with preterm delivery, as well as the association between the presence of pathogenic oral bacteria in the placenta and adverse pregnancy outcomes. On the other hand, some previously published papers found periodontal bacteria in placentas of term pregnancies. In spite of a huge research done on the topic, both experimental and clinical, there are many controversial opinions about the role of periodontal infections in preterm birth. Thus, this comprehensive review addresses this very important topic and evaluates novel strategies of preventive and therapeutic approaches.

## 1. Introduction. Preterm Labor

### 1.1. Definition and Epidemiology

Preterm labor is defined as a birth before 37 weeks of gestation and occurs in 5–20% of pregnancies [1,2]. It is a leading cause of newborns’ morbidity and mortality, and the second cause of childhood death before the age of 5 years [1,3]. According to data, in the USA the preterm delivery rate is 12–13%, whereas in Europe and other developed countries is up to 9% [4,5]. It is estimated that around 15 million preterm neonates are born annually with the highest rates in Africa, South Asia and North America.

Preterm births account for almost 75 percent of perinatal mortality and more than 50 percent of the long-term morbidity [1,3,5,6,7]. The most common neonatal complications include newborn respiratory distress syndrome (RDS), neural system injury, necrotizing enterocolitis, neonatal jaundice, and infections [2,3]. All the above-mentioned complications lead to prolonged hospitalization, which in turn increases the risk of hospital-acquired infections and death.

### 1.2. Etiology and Risk Factors

Preterm labor is a heterogeneous condition of multifactorial origin influenced by maternal, fetal and environmental factors [8]. Due to the multiple etiologies and risk factors involved, the prediction of preterm labor remains challenging. There are many maternal and/or fetal characteristics associated with preterm birth, including maternal demographic and nutritional status, parity and current pregnancy history, length of the uterine cervix, addictions, infection, and genetic markers (Table 1) [5,7,8]. The process of preterm labor is thought to be initiated by multiple mechanisms, including infection, immunologically driven processes, placental ischaemia, uterine over distension, bleeding, and other actors [1,5,9,10]. Assuming that many factors might be involved in each particular preterm birth, a precise mechanism cannot be identified in most of cases. 

The goal of this review is to evaluate the existing literature sources to identify the role of periodontal infection in the onset of preterm labor. Early identification of pregnancies at highest risk for preterm labor may help in the improvement of the existing and development of new therapeutic management options. It also could serve to improve strategies to prevent neonatal morbidity associated with preterm birth.

## 2. Materials and Methods

A non-systematic review was performed through a search on the following databases: PubMed, EMBASE, The Cochrane Library (Cochrane Database of Systematic Reviews, Cochrane Central Register of Controlled Trials, Cochrane Methodology Register), and Web of Science, research registers (such as www.cliniclatrials.gov, accessed on 29 August 2020). We used the medical subject heading (MeSH) term Premature Birth (MeSH Unique ID: D047928) in combination with Periodontal Abscess (MeSH Unique ID: D010508) and selected papers published in English, with no time restrictions about the year of publication. Titles and abstracts of studies retrieved using the search strategy, and those from additional sources, were screened independently by two review authors (M.T. and G.A.) to identify studies that potentially meet the objectives of this non-systematic review. The full text of the eligible articles was retrieved and independently assessed by other two review team members (S.T. and G.B.). Any disagreement between them over the eligibility of particular articles was discussed and solved with a third collaborator (A.S.L.). Two authors (G.A. and M.R.) independently extracted data from articles about study characteristics, populations, type of intervention and outcomes. Due to the nature of the findings, we opted for a narrative synthesis of the results from selected articles.

## 3. The Link between Infectious Pathogens and Preterm Labor

### 3.1. Infectious Pathogens as a Cause of Preterm Labor

There is very strong evidence that infection plays a major role in the pathogenesis of preterm labor. Studies suggest that infection may be responsible for 25–40% of preterm birth cases [5,9,11,12]. The relationship between infection, inflammatory response and preterm labor has been confirmed by multiple findings in preterm labor patients including intrauterine/intra-amniotic and extrauterine maternal infections/inflammations: vaginal infections, urinary tract infections, pneumonia, and periodontal disease [7,12,13,14,15,16,17,18]. The link between infection and preterm birth could also be supported by the fact that antibiotics administered in asymptomatic bacteriuria prevents preterm birth [1,19,20]. 

Multiple infectious agents could cause the vaginal infection and later via the above-mentioned routes the intrauterine/intra-amniotic infection: *Escherichia coli*, *Enterobacter*, *group B Streptococcus* (GBS)/*Streptococcus agalacteae*, *Staphylococcus epidermidis*, *Chlamydia trachomatis*, *Micoplasma hominis* and *Ureaplasma urealiticum*, *Neisseria gonorrheae*, *Treponema pallidums*, *Trichomonas vaginalis*, HIV, Hepatitis B and C, as well as bacterial vaginosis (BV). Some of them can be easily ruled out while others might remain undetected and a silent cause of infection.

The pathogens most commonly reported in the amniotic fluid are genital *Mycoplasma spp.*, and, specifically, *U. urealyticum* [1,5,7]. Women who are tested and found to be positive for *U. urealyticum* often have spontaneous preterm labor or preterm premature rupture of membranes (PPROM) [5,7,21]. Importantly, the earlier the gestational age at preterm labor, the higher the frequency of intrauterine infection [1,5,22]. Many researchers reported the role of BV [5,13,23,24]. Although the role of BV itself remains largely unknown, a strong risk for preterm labor was confirmed [5,13,23]. BV has shown to be a cause for spontaneous abortions, highlighting the role of genital infection for adverse pregnancy outcomes (APO) [13]. 

It is not always clear whether other genital infections like *Trichomonas vaginalis*, *Treponema pallidum*, *Neisseria gonorrheae,* etc. are responsible for preterm birth [1,5,25]. *Trichomonas vaginalis* seems to be responsible for some preterm labor cases with a relative risk (RR) of about 3 [5]. Conversely, *Treponema pallidum*, *Neisseria gonorrheae* and *Chlamidia trachomatis* are reported to be associated with preterm birth only in the presence of a maternal immune response with RR of 2 [5,26]. Moreover, women with genital infections usually have other risk factors, and many studies have not considered concurrent variables.

Vaginal infections have been found to be linked with an increased risk of PPROM and preterm birth [5]. Being identified as a cause of vaginal infection, microbes access the uterine cavity by an ascending route from the vagina and the cervix [1,5,10]. In turn, intrauterine infection has been identified to be a frequent cause of preterm labor through the spread to the amniotic cavity [1,5]. There are quite a few other routes of its propagation: direct implantation at the time of invasive procedures, retrograde spread through the fallopian tubes, and haematogenous dissemination through the placenta [5,9,21]. 

Infections of other sites, non-genital, like urinary tract infections, pneumonia, and periodontal infection, are also linked to the preterm labor [5,10]. Some studies suggest an increased risk of preterm labor in periodontal disease [5]. One potential explanation for the relation is that gingival microbes through the bloodstream could reach the uterine cavity and the placenta, resulting in an intra-amniotic infection [5,7,18]. However, the mechanism behind the link between periodontal pathology and preterm labor still remains unclear in some details.

### 3.2. Pathophysiological Mechanisms 

There are two major pathways in biological mechanisms of APO related to oral pathogens defined by the consensus report from the joint European Federation of Periodontology/American Academy of Periodontology workshop on periodontitis and systematic diseases [9,27]: (1) direct mechanisms—oral microorganisms invade the placenta and amniotic cavity via hematogenous dissemination, or in an ascending route via the genitourinary tract; (2) indirect mechanisms, promoted by inflammatory mediators produced in periodontal tissues, in response to the pathogens invasion. These mediators may directly affect the fetal-placental unit or circulate to the liver and increase the systemic inflammatory response, which could later affect the fetal-placental unit [5,7,28].

Vaginal infections lead to the increased concentration of the inflammatory markers that can be identified in cervical and vaginal secretions and have been shown to be significantly associated with the preterm labor rates [9,13,29]. Those include interleukin (IL)-6, IL-8, IL-1β, ferritin and tumor necrosis factor α (TNFα) [5,13]. Endotoxins released by the microorganisms together with the proinflammatory cytokines stimulate the production of prostaglandins (PGs), other inflammatory mediators, and matrix-degrading enzymes. PGs in turn stimulate myometrium leading to increased uterine contractility and onset of preterm labor and PPROM [1,5,7,9,13]. 

## 4. Periodontal Pathogens and Pregnancy

### 4.1. Anatomical and Physiological Background

#### 4.1.1. Gingival Changes in Pregnancy

Women’s bodies undergo important adaptations in many organ systems and hormonal changes during pregnancy. As well as the other systems, gastrointestinal tract and the oral cavity as a part of it are also under this influence. In this period increased sensitivity to stimuli occurs in the gingiva [30]. Vomiting can negatively affect oral hygiene or may cause erosions in the oral cavity [30]. The following oral conditions have been described as affecting pregnant women to a greater degree than their non-pregnant counterparts: dental caries, gingivitis, pregnancy granuloma, and periodontitis [31]. 

Gingivitis is inflammation of the superficial gum tissue [32]. Gingivitis is the most frequent oral disease in pregnancy, with a prevalence of 40–75% according to different sources [30,31,32]. Approximately 50% of women with preexisting gingivitis will face significant exacerbation during pregnancy due to changes in estrogen and progesterone levels combined with oral microbiota alterations and pregnancy-related physiologic immunodeficiency [30,31,32]. Pregnancy gingivitis could be seen very often and usually starts at the first trimester of gestation, worsens as the pregnancy progresses before reaching a peak close to the end of the third trimester and heals spontaneously after birth [30,31,32]. However, in the last weeks of gestation, rates of gingivitis usually decreases and immediately in the postpartum period, the gingival tissues are found to be comparable to those seen during the first trimester of pregnancy.

#### 4.1.2. Factors Influencing Gingival Changes

The decline and exacerbation in oral health during pregnancy depends on multiple factors [30]. During the first trimester of pregnancy, some women may have eating behavior changes like increased consumption of carbohydrates or even pica. Pregnant women gingiva bleeds more readily due to the elevated concentration of estrogens, therefore women may avoid brushing their teeth. However, the exact role of these hormones on the increase of gingival inflammation occurrence and deterioration was speculated [32]. Therefore, oral care becomes more important in pregnancy. Recently, a published paper associated gingival changes in pregnancy with increased vascularization and blood flow in conjunction with the pregnancy-related physiologic immunodeficiency and changes in connective tissue metabolism [30,31,32].

As mentioned before, vomiting, especially during the first trimester of pregnancy, increases the acidity in the mouth. Because episodes of vomiting in cases of hyperemesis gravidarum are usually very frequent, the pregnant woman may not pay enough attention to oral care after each event [30,31]. Subsequently, if the teeth are not brushed, an acidic environment will develop in the mouth and support some pathogens overgrowth. Vomiting and dehydration will lead to decreases of the oral saliva flow and contribute to the periodontal problems as well. For these reasons, the rate of caries increases in the first trimester of pregnancy. Due to the existence of the listed risk factors, it is important to draw more attention to dental care and health during this period.

### 4.2. Relationship between the Presence of Periodontal Pathogens in the Oral Cavity and the Preterm Labor 

For the past 30 years, it has been proposed that oral pathogens could act as an infectious reservoir and interfere with pregnancy outcomes. The first association between periodontal disease and preterm labor was investigated and reported in 1996 by Offenbacher [33]. In later studies, it has been confirmed that approximately 40% of pregnant women have some form of periodontal disease, and the rate is higher among women of low socioeconomic status [34]. The most common maternal oral diseases that potentially could lead to APO due to infection dissemination include dental caries, gingivitis, and periodontitis [18,35,36]. Multiple studies evaluating the link between periodontal pathologies and preterm labor, low birth weight, and preeclampsia, have been published using case-control, cohort, and cross-sectional study designs. However, some studies failed to confirm these associations [37]. From the other side, a strong relationship between preterm birth and periodontitis was confirmed in recently published study, suggesting that oral infections may be considered a risk factor for gestational adversities [38].

#### 4.2.1. Definition, Epidemiology and Classification of Periodontal Disease

Periodontal diseases represent one of the most common chronic infections in humans with a prevalence of 10 to 60% among adult population [9]. Periodontal diseases include many different inflammatory conditions that affect the gingiva, but also the alveolar bone and the periodontal ligament that anchors the tooth to the bone. 

Periodontitis is a chronic multifactorial inflammatory disease associated with dysbiotic plaque biofilms and characterized by progressive destruction of the tooth-supporting tissues [39]. It is a relatively common clinical condition, which occurs in more than 30% of people in some populations [39]. The prevalence among pregnant women ranges between 5% and 20% [39]. 

Periodontitis has been classified by different ways: (1) based on stages defined by severity, complexity and extent and distribution (stage I, II, and III); (2) based on grades that reflect biologic features of the disease including evidence of, or risk for, rapid progression, expected treatment response, and effects on general health (grade A, B, and C) [39]. There are separate classifications of necrotizing periodontal diseases, endo-periodontal lesions, and periodontal abscesses [39].

#### 4.2.2. Pathophysiologic Mechanisms of Periodontal Disease 

Periodontal pathology usually begins with a localized inflammation of the gingiva, called gingivitis, caused by dental plaques, microbial biofilms that form on the teeth and gingiva. If left untreated, the inflammation in gingivitis can lead to periodontitis [40]. The inflammatory response in periodontal diseases is found to contribute to the development of certain systemic diseases such as Type II Diabetes mellitus [41], cardiovascular diseases [42,43], rheumatoid arthritis [44], and even oral cancer [45].

Periodontal disease initiates as infection caused by an overgrowth of certain bacterial species in the subgingival sites. However, most of the periodontal tissue destruction is caused by the persistent inflammation that develops as a host response [9,10,46]. The multispecies subgingival biofilm that causes periodontal disease consists mostly of Gram-negative, anaerobic bacteria. The periodontal bacteria are divided into complexes or clusters. In the early stages of biofilm formation, the bacteria that are present mostly belong to the “blue”, “green”, “yellow” and “purple” clusters. More pathogenic bacteria of the “orange” cluster appear as the biofilm matures, including *Campylobacter rectus*, *Fusobacterium nucleatum*, *Peptostreptococcus micros*, *Prevotella intermedia* and *Prevotella nigrescens*. The biofilm is further colonized by the bacteria from the “red” cluster that are more aggressive, including *Porphyromonas gingivalis*, *Tannerella forsythia* and *Treponema denticola*. Although many virulence factors of the pathogenic bacteria are already known, the exact contribution of each species in the development of periodontitis is still unknown [46]. 

However, microbial colonization and biofilm formation is just the initial step in the periodontal disease development. The disease develops when the host’s immune system overreacts to the presence of bacteria, a process called dysbiosis [47]. This imbalance is very complex, there are great variances both of the biofilm composition and the host immune reaction profiles which lead to tissue damage due to a heightened inflammatory state [48]. The alveolar bone is resorbed by osteoclasts, ligament fibers are degraded by enzymes called matrix metalloproteinases, and granulation tissue is formed [49]. This situation leads to the loss of tooth if it is not treated in time. 

#### 4.2.3. The Link of Periodontal Disease in Pregnancy and Preterm Birth: Studies Contradictions 

Approximately, in 50% of cases of premature birth the etiological factors are known and infectious agents responsible for almost 75% of them. However, the other half of cases remain idiopathic [50,51,52]. 

During pregnancy, due to hormonal changes, there may be a tendency towards development of periodontal disease. In particular, there is an increase of anaerobic gram-negative bacteria such as *Fusobacterium nucleatum*, *Treponema denticola*, *Tannerella forsythia*, *Campylobacter rectus*, *Eikenella corrodens*, and *Selenomonas sputigena* [50,51] that could contribute to the incidence of periodontal pathology. 

Oral infections might be considered as one of the factors contributing to the preterm labor incidence, since commensal bacterial species of the oral cavity were found to be disseminating to the fetoplacental unit of women with term gestation and APO [50,53]. The following microbes have been found to be strongly associated with APO: *Fusobacterium nucleatum*, *Campylobacter rectus*, *Porphyromonas gingivalis*, and *Bergeyella spp.* [27,50].

Many investigations have shown an association of periodontal disease, prematurity, and low birth weight [18,36,38]. On the other hand, clinical trials have studied the effect of periodontal treatment on APO and showed controversial results [50,54,55]. There are at least two explanations for such contradictory findings: (1) colonization of fetoplacental site by periodontal pathogens appears at the end of the first trimester and its detrimental effect cannot be completely eliminated by antimicrobials, and (2) significant diversity between investigations in regard to the populations’ race, age, and the periodontitis definition accepted for these studies [40,56].

The conclusions of many studies/systematic analyses could be contradictory due to the relative heterogeneity of studied populations according to ethnicity, different risk factors discussed, diversity in socio-economic and education levels, and periodontal status definition.

### 4.3. Development of the Periodontal Disease in Pregnancy

During pregnancy, periodontal status changes. Due to the physiologic changes in the immune system, pregnant women are more prone to have inflammatory conditions and increased rate of gingival bleeding. It appears that pregnancy might lead to an increase of periodontal disease severity in women suffering from periodontitis before pregnancy [9,10,22,55]. 

Pregnancy related nausea and vomiting are relatively frequent in early pregnancy. As a consequence, gastric acid would damage periodontal tissue barriers to various pathogens, which could be responsible for placental infection and systemic inflammation leading to preterm birth [57].

Furthermore, hormonal fluctuations during pregnancy have been proposed to initiate changes in the composition of oral biofilm leading to exacerbation of gingival inflammation. Researchers discuss that hematogenous dissemination of microbes and pro-inflammatory mediators from sites of periodontal infection into the placenta, fetal membranes, and amniotic cavity induces pathological processes that lead to APO [58]. However, researchers’ opinions remain controversial as some studies supported this hypothesis, but the others failed to demonstrate improved perinatal outcomes following treatment of periodontal disease in pregnancy [59].

**Figure 1:**
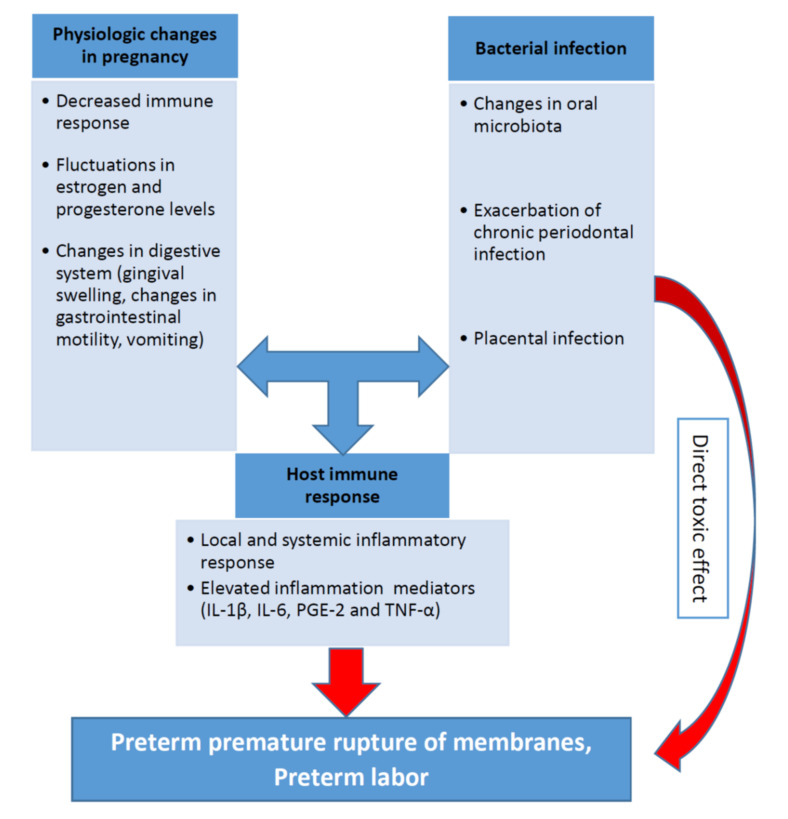
Role of periodontal infection in preterm labor.

The biological plausibility of association between periodontal disease and APO is based in a hypothetical model of two major pathways (direct and indirect) responsible for the pathologic process [7,60,61,62]. It was discussed in one of the previous sections related to the pathophysiological mechanisms. The pathogenesis of periodontal inflammation and preterm birth including the role of the main inflammatory mediators (IL-1β, IL-6, PGE-2 and TNF-α) are schematically presented on the Figure 1. 

Relationship between pro-inflammatory cytokines, periodontal infection, and preterm birth is recognized in both experimental conditions and animal models. Most animal models show evidence of the causal link between APO and periodontal infection. In a study by Ao et al. (2015), *P. gingivalis* induced preterm birth and low birth weight in pregnant mice and significantly elevated maternal levels of circulating TNF-α, IL-17, IL-6 and IL-1β [63]. Moreover, immunohistochemical analysis detected colonization of *P. gingivalis* in the placenta of *P. gingivalis*-infected pregnant mice [63]. Some studies found that gram-positive and gram-negative intracellular bacteria can be present in the basal layer of the placenta. It is also suggested that presence of pathogenic oral bacteria in the placenta is related to APO [58]. On the other hand, periodontal bacteria have been found in normal placentas without APO [64]. Another possible mechanism is that inflammatory mediators produced by infected periodontal tissue affect the fetoplacental unit and myometrium. In periodontal diseases, pro-inflammatory cytokines and mediators, including IL-1β, IL-6, TNF-α and PGE2, were produced in subgingival area and then entered systemic circulation [5,7,65]. 

Some authors identified periodontal disease as the cause of more than 18% of all preterm birth cases. Therefore, there is a significant evidence that periodontal pathogens, its enzymes and toxins can induce inflammation in placental tissues and cells [66,67]. Thus, investigations of the association between periodontal disease and APO are highly relevant for clinical medicine [68,69,70]. 

## 5. Possible Management

### 5.1. Dental Management during Pregnancy

There is no evidence-based effective guideline developed for periodontal infection treatment in pregnancy. In general population the aim of periodontal treatment is to reduce periodontal tissues infection through a careful and continuous oral hygiene education, medical and a mechanic (surgical) treatment. In the severe chronic or aggressive forms of periodontitis the treatment includes the administration of systemic antibiotics [39]. 

Pregnancy related guidelines on oral care varies from country to country. In some countries, recommendations on oral care and treatment are included in the pregnancy follow-up guidelines [71]. In the USA, only up to 34% pregnant women consult a dentist during their pregnancy [10]. Pregnant women, obstetricians and dentistry specialists are prudent while prescribing a specific dental treatment to avoid other possible pregnancy complications. Moreover, around 50% of obstetricians rarely recommend a dental examination unless it is suggested by country-specific guidelines [10]. Even if pregnant women are referred to dentists, only 10% of the specialists perform all necessary treatments, and 14% of dentists are against using local anesthetics during pregnancy [10].

A meta-analysis by Cochrane Oral Health Group has assessed the effects of treating periodontal disease in pregnant women in order to prevent or reduce perinatal and maternal morbidity and mortality [72]. There were 15 RCTs including 7161 participants analyzed. The researchers compared periodontal treatment versus no treatment during pregnancy and periodontal treatment versus alternative periodontal treatment [72]. The meta-analysis shows no clear difference in preterm birth <37 weeks (RR 0.87, 95% CI 0.70 to 1.10; 5671 participants; 11 studies; low-quality evidence) between periodontal treatment and no treatment [72]. The authors concluded that it is not clear if periodontal treatment during pregnancy has an impact on preterm birth. There is insufficient evidence to determine which periodontal treatment is better in preventing adverse obstetric outcome [72]. On that basis, future research should be performed to report periodontal outcomes alongside obstetric outcomes.

In the Obstetrics and Periodontal Therapy (OPT) randomized controlled trial performed almost 15 years ago, periodontal treatment was not found to be associated with the increased risk of preterm labor [73]. Contrary to that, treatment of periodontal disease before 21 weeks of gestation was found to prevent preterm births [74]. Despite controversial reports, the majority of studies concluded that periodontal treatment in pregnancy is safe and can improve periodontal status. Therefore, the early diagnosis and management of periodontal disease should be recommended. It will help to decrease the impact of periodontal disease on preterm birth and APO incidence. The preventive oral care and pre-pregnancy management is the best way to prevent oral diseases and their effect on pregnancy. In case of periodontal disease diagnosed in pregnancy, a frequent monitoring of the patient should be suggested to control the disease and plan appropriate intervention that will decrease the preterm birth risk. For severe periodontitis, systemic antibacterial treatment with metronidazole or amoxicillin could be prescribed in complex with mechanical treatments. The majority of the available pieces of evidence demonstrate no deleterious effect of antibiotic use on pregnancy outcome especially for metronidazole [75,76].

Published guidelines on oral care during pregnancy highlight that it is an underdeveloped area and the relevance of oral hygiene during pregnancy is insufficiently acknowledged by dentistry and obstetric specialists. Recently published overview of systematic reviews demonstrates strong evidence for a link between periodontal disease and APO [10,36,37,55]. 

### 5.2. Treatment Modalities Based on the Gestational Age

The most important steps of fetal organogenesis take place in the first 12 weeks of gestation and is very sensitive to the influence of external factors, especially drugs. During the first trimester, only emergency dental treatment is indicated [39]. Oral hygiene should be reinforced with plaque control done by the dentist. The second trimester is the safest period for elective dental treatment if necessary [31,39]. Despite the fact that some treatment options are available during pregnancy, it is preferable to postpone extensive reconstructions or major surgical procedures after delivery [10,39,77].

Regarding the systemic treatment with medications/antibiotics during pregnancy, the main concern is the possible teratogenic effect that might appear. Before prescribing a drug to a pregnant patient, the dentistry specialists follow the Food and Drug Administration (FDA) classification for the prescription of drugs to pregnant women based on the risk of adverse fetal outcomes [39,78].

### 5.3. The Importance of Oral Care before Conception

As recommended by the recent guidelines, it is appropriate to undergo dental examination as a part of preconception counselling in order to prevent dental and periodontal diseases [39]. The main purpose and aim of prevention are to reduce the presence of bacterial plaque through motivation to proper daily oral hygiene, education in proper nutrition, a balanced diet, and low intake of sugars, and, finally, professional dental hygiene procedures. For these reasons, close interdisciplinary collaboration between obstetricians and dentists is very important [31,39]. Later, after delivery, the patient may undergo certain dental visits and dental treatment, without any risk [39]. Appropriate dental care and prevention during pregnancy may decrease number of risk factors, and reduce poor prenatal outcomes [39,78]. Oral-associated bacteria are present in vaginal region, in the specific context of interpersonal relationships [29,79]. Therefore, interventions in oral cavity and management of periodontal pathogens are very important for both partners, not before conception for women only. 

### 5.4. Future Tasks for Gynecologists and Dentists 

Having the global burden of periodontal disease and the range of APO that have been associated with it, there is still a need to describe the role of local periodontal bacteria in the placenta and oral cavity in relationship to local and systemic inflammatory cytokines for the pathogenesis of preterm labor. Additionally, from the public health point of view, identification of the underlying mechanisms will enable development of preventive strategies aimed at reducing of preterm birth, as the most important APO. Thus, there is a need to establish a new preventive and therapeutic approaches at earlier stages of pregnancy, perhaps even for the period of preconceptional counselling.

According to some authors, appropriate, timely and adequate periodontal treatment performed during pregnancy appears to be safe for the mother and the fetus [10,52]. It may greatly contribute to the proper control and decrease the rate of periodontal infection and, therefore, reduce the risk of preterm birth. On the other hand, if periodontal treatment is done late and not able to control periodontal inflammation, it appeared to increase the risk of preterm birth as a result of systematic error [22,52]. Therefore, education of healthcare professionals, both gynecologists and dentists, patient information and up-to-date guidelines on oral care would greatly impact the periodontal disease burden and preterm labor rate attributed to it.

Future studies that will aim to confirm the positive association between periodontal disease and preterm birth should utilize a more thorough methodology, including the appropriate definition of the condition, adequate control of confounders for preterm labor, and application of effective interventions to eliminate periodontal infection [80]. 

## 6. Conclusions

Multiple studies provide strong evidence role of the periodontal infection in preterm labor. Furthermore, conclusions of many studies reported that periodontal pathogens can be transferred from periodontal tissues to the feto-placental site and cause an infection. However, the exact mechanisms by which inflammation disseminated from the oral cavity to the placenta contribute to adverse pregnancy outcomes remain unclear and require further investigation. Moreover, there are many pieces of evidence supporting the importance of the establishment a prevention program to minimize problems during pregnancy. Prevention means scheduling the preconception counselling visits, timely examinations and reducing the presence of bacterial plaque, through professional hygiene sessions, education, motivation to proper oral hygiene and nutrition. For these reasons, it is essential to develop a more effective interdisciplinary collaboration between obstetricians and dentistry specialist in order to achieve an optimal health for pregnant women.

## Figures and Tables

**Table 1:** Risk factors associated with spontaneous preterm birth.

Risk Factors	OR	95% CI	Reference
Second trimester cervical length ≤2.50 cm	6.9	4.3–11.1	[8]
Vaginal bleeding in third trimester	5.9	5.1–6.9	[6]
Short interval between pregnancies (<12 months)	4.2	3.0–6.0	[6]
Previous preterm birth with a single newborn	2.62	1.99–3.44	[8]
Vaginal bleeding in first trimester	2.0	1.7–2.3	[6]
Periodontal disease	2.0	1.2–3.2	[6]
Prior cervical conization	1.7	1.24–2.35	[6]
Age younger than 18	1.7	1.02–3.08	[6]
Low socioeconomic condition	1.66	1.06–2.61	[8]
	1.75	1.65–1.86	[6]
Pregnancy with male fetus	1.51	1.02–2.24	[8]
Asymptomatic bacteriuria	1.5	1.2–1.9	[6]
Bacterial vaginosis	1.4	1.1–1.8	[6]
Family history of preterm birth	1.35	1.12–1.63	[8]
Maternal smoking	1.27	1.21–1.33	[8]
	1.7	1.3–2.2	[6]

## Data Availability

Not applicable.
